# Age- and sex-dependent associations between the number of older siblings and early-life survival in pre-industrial humans

**DOI:** 10.1098/rspb.2025.1525

**Published:** 2025-09-03

**Authors:** Mark Spa, Euan A. Young, Virpi Lummaa, Erik Postma, Hannah L. Dugdale

**Affiliations:** ^1^ Groningen Institute for Evolutionary Life Sciences, University of Groningen, Groningen, The Netherlands; ^2^ Department of Biology, University of Turku, Turku, Finland; ^3^ Centre for Ecology & Conservation, University of Exeter College of Life and Environmental Sciences, Penryn, UK

**Keywords:** family evolution, human life history, cooperation, sibling rivalry, parental investment, optimal brood size

## Abstract

Siblings are an important part of an individual’s early-life environment and may therefore play an important role in shaping an individual’s survival. The quantification of sibling effects on survival is challenging, however, especially in long-lived species with extended parental care and overlapping generations, such as humans. Here, we use historical parish data from Switzerland to quantify how the number of older siblings and their survival status, age and sex are associated with childhood survival. Across 2941 focal individuals born between 1750 and 1870, the total number of older siblings did not predict an individual’s childhood survival probability. However, distinguishing between siblings by their survival status, age and sex revealed several associations, which in some cases also interacted with the sex of the focal individual: while older brothers close in age reduced the survival of girls (but not boys), having more older sisters close in age improved their younger sibling’s survival. Our results therefore suggest that older siblings play an important role in shaping early-life survival and highlight that the strength and direction of sibling-related associations are context-dependent and can arise through both biological and cultural factors.

## 1. Introduction

How the early-life environment shapes individual variation is of keen interest to evolutionary ecologists [1,2]. Across a variety of animal species, older siblings are one important aspect of the early-life environment [3,4], with the potential to positively or negatively shape evolutionarily and demographically important traits, including survival and reproduction [5]. However, quantifying the effects of siblings on offspring fitness components has proved challenging, especially in species with long, slow life histories and overlapping generations [4].

Whether siblings provide benefits or compete depends upon a species’s life history and degree of cooperation. When siblings do not act cooperatively, having more siblings will lead to competition because a limited amount of resources, such as parental care, is split among more offspring [6,7]. In humans, this is referred to as the resource dilution hypothesis [8], which posits that parental resources are finite, and when they are divided among more children, this reduces the benefits available to each child. However, siblings may vary in their ability to compete for these finite resources, generating variation in how siblings affect each other. For example, competitive abilities can vary between the sexes in species who are sexually dimorphic [9]. Furthermore, in species where offspring are not produced simultaneously, older siblings will be more developed and have a competitive advantage over younger siblings [10]. Additionally, even when parents aim for equal distribution of resources among their offspring, later-borns may be at a disadvantage because they enter a family when resources are already depleted [11]. Hence, in addition to the number of siblings, the birth order and age of siblings are additional determinants in the resource dilution hypothesis. To complicate matters further, negative sibling interactions through competition may be offset by the positive effect of cooperation, promoted through kin selection [12,13], including direct help or behaviours that increase shared familial resources [14]. Thus far, ecological studies have largely focused on within-brood sibling interactions in birds [4], and understanding the balance of cooperative and competitive sibling interactions across sexually dimorphic species with multiple reproductive events is the subject of ongoing research (see [15] for research on elephants and [16] for research on humans).

In humans, sibling effects have attracted interest from researchers from different fields, studying a variety of cultures and traits, including dispersal behaviour [17–19], nutritional status [20,21], educational attainment [8,22] and marital timing [23]. Some studies have also looked at fitness outcomes, including childhood survival, in pre-industrial societies [16,19,24,25]. In these human societies, short interbirth intervals combined with long development times [26,27] result in parents raising multiple dependent children simultaneously [28,29]. While humans are a highly cooperative species, older siblings who are closer in age and still depend on their parents might compete with younger siblings and negatively impact their development [30]. This could occur through direct competition for parental resources or via effects on the health and condition of the mother during previous pregnancies [31,32]. Conversely, it is expected that older and more independent siblings (often defined as being at least 5 years older; e.g. [33]) can have positive effects on survival through cooperative behaviours [30]. These behaviours can provide not only direct benefits, such as taking on childcare duties [28], but also indirect benefits, because older siblings take over tasks that allow parents to focus on caring for newborns [33] or increase familial resources (e.g. through foraging, agricultural labour [28,34] or paid labour in industrialized societies [35]). Although isolating specific mechanisms is challenging, studies have generally found that having more older siblings close in age is associated with lower early-life survival [33], while having more older siblings further in age is associated with higher early-life survival [36,37].

Biologically and culturally mediated sex differences [38] can interact with sex differences in development time to make sibling interactions in humans highly sex-dependent [39]. Biologically, because males are on average larger and require more resources [40,41], the effect of older brothers on their younger siblings’ survival could be expected to be more negative compared to the effect of older sisters [42]. However, males also have lower childhood survival rates than females, perhaps due to their weaker immune system [40,43,44], and the death of a previously born older sibling may decrease their impact on their younger siblings [31], Hence, the survival status of older brothers can moderate their effect on survival, and in addition to age differences, both the sex and the survival status of siblings are important to account for in studies.

The negative biological effects of having older brothers can be moderated by cultural factors, which could explain why several studies found no sex-specific effects of older siblings on early-life survival [36,37,45]. For example, having more older brothers enhanced survival to 15 years in pre-industrial Finland, possibly because they made economic contributions [19]. On the other hand, in cultures where only sisters provide help to younger siblings, the number of older sisters positively correlates with survival [46]. Finally, in cultures with a preference for sons (e.g. due to patrilineal inheritance), sisters may be more negatively affected by the presence of brothers [33]. This may happen because more resources are allocated to males rather than because of direct harm by parents or siblings [47,48]. This aligns with studies arguing that resource dilution is not uniform but conditional, leading to the development of the gendered and context-dependent resource dilution hypothesis [49,50]. Regardless of the mechanism, these effects can be so strong that they reverse the biological differences in childhood mortality between sexes [51]. Son preference can, in turn, increase the competition for resources among sisters, placing younger female siblings at an even greater disadvantage [52]. When this combines with older brothers bringing in more resources, it can feed into a wider picture of same-sex competition but opposite-sex benefits [16,33,53]. It is thus crucial that studies account for age differences, survival statuses and how they interact with sex when examining the effects of siblings on early-life survival.

Few studies have simultaneously considered both siblings who may compete for resources and those who may provide support (but see [33]). Here, we aim to fill this knowledge gap and examine evidence for cooperative and competitive interactions between siblings shaping childhood survival to age 5, an important factor in the evolutionary and demographic history of humans [54]. To this end, we use historical life-history data from 2941 individuals born in the period 1750−1870, adapted from Swiss church parish records [55]. In this population, both fertility and childhood mortality were high during this period, making the population particularly valuable for investigating the effect of siblings on childhood survival patterns. We first quantify the association between childhood survival and total number of older siblings while controlling for potentially important confounders, such as grandparental presence [56], parental presence [57], parental age [58] and socioeconomic status [59]. We then conduct a decomposition of the number of older siblings in a series of models, separating siblings by whether they had died before the birth of the focal individual (their survival status), whether they were born close (< 5 years) or far in age (≥ 5 years) from the focal individuals, and whether they were sisters or brothers. We estimate the associations between childhood survival and the number of older siblings in each of these categories, while allowing for these associations to be dependent on the sex of the focal individual. On the whole, these analyses provide a uniquely detailed insight into how siblings may shape child survival and how this may be modulated by cultural factors such as parental preferences or gender-specific roles within the family.

We predict that the number of older siblings who are at least 5 years of age is associated with an increase in childhood survival because the positive effects of helping outweigh the negative effects of competition. Conversely, we expect the number of older siblings who are under 5 years of age to be associated with decreased childhood survival, owing to maternal health and/or competition for parental resources. We also expect associations with older siblings to vary based on the sex of both the older siblings and the focal individual and predict brothers to be associated with decreased childhood survival owing to larger resource requirements. Finally, a son preference would manifest itself as an interaction between the sex of the sibling and the sex of the focal individual, with the association between the number of older brothers and childhood survival being more negative (or less positive) for females than for males.

## 2. Methods

### (a) Study population

We used data from an extensive genealogical archive [55] that covers two parishes situated on the Swiss Alpine plateau: Linthal (46°55′ N, 9°00′ E) and Elm (46°55′ N, 9°10′ E). This archive contains birth, marriage and death dates for individuals (including unbaptized and stillborn individuals) born between 1540 and 1998, but 73% were born after 1800. For 96% of the individuals, both their birth date and the identity of their parents were known, allowing for the characterization of family structure at birth. Although the precise death date was missing for 41% of individuals, this was mainly due to emigration as an adult, and deaths before the age of 5 were unlikely to have been missed.

We limit our analyses to individuals born between 1750 and 1870 as sample sizes for earlier years were relatively small (e.g. < 30 recorded births per year), and after 1870 early-life survival gradually improved in Switzerland [60]. Our data show that during this period, the median lifespan was 31, and survival from birth to age 5 (childhood survival) was relatively low (67%). These values are broadly consistent with historical estimates from other eighteenth- and nineteenth-century European populations [54,61]. At the same time, our data show that fertility was high, with a median of five children per reproductive woman, ranging from 1 to 22. Hence, this can be considered a stage 1 demographic transition (i.e. pre-industrialized) population [62,63]. During this period, the population is furthermore representative of a northern or western European population, with relatively late ages-at-first birth (median age 25) owing to the wealth accumulation that was required pre-marriage [64]. During this period, the region can be considered pre-industrial, as by 1850 around 50% of the Swiss population was agricultural [65], and in our data, 40.6% of children had a father who worked in the agricultural sector (*n* = 3021/7439). Other common areas of occupation for fathers were the military (8.5%), construction/carpentry (7.8%), administrative/clerical (6.9%) and factory work (3.5%).

### (b) Childhood survival

We treat childhood survival as a binary variable defined as survival until age 5 [33,66,67]. Survival was determined for all individuals who had a recorded birth and death year. This included 194 individuals who died on the day they were born. Individuals with an unknown year of death that were known to have married and/or reproduced were assumed to have survived beyond the age of 5 (*n* = 2328). Overall, childhood survival status could be determined for 93% of the individuals with a known birth date (*n* = 11 878).

### (c) Sibling classifications

Using the identity of their parents, we grouped individuals into nuclear families. Limiting ourselves to full siblings of parents that only married once, we counted the number of older siblings at an individual’s birth, which ranged from 0 to 15 older siblings. In addition to the total number of older siblings, for all older siblings with known birth and death dates, we determined whether they survived until the birth of the focal individual (survival status; living older siblings range = 0–11, deceased older siblings range = 0–8). We also determined whether alive older siblings were less than (range = 0–4) or at least 5 years (range = 0–10) older than the focal individual (hence < 5 or ≥ 5, respectively). These categorizations aimed to distinguish between siblings that, for the majority of the focal individual’s first 5 years, were unlikely (< 5 years older) or likely (≥ 5 years older) to have been able to provide benefits (see [33,36]). These may include not only direct help or care but also the contribution to family wealth through labour [68]. Finally, we distinguished between male and female older siblings in each age category (i.e. brothers and sisters) (brothers < 5 older: range = 0–4; brothers ≥ 5 older: range = 0–7; sisters < 5 older: range = 0–3; sisters ≥ 5 older: range = 0–6).

### (d) Focal individual characteristics

For each focal individual, their sex was recorded (males: *n* = 6567; females: *n* = 6243) to account for factors such as the higher susceptibility of males to mortality during early life and a possible son preference [43,44,47]. This also allowed for the examination of sex-dependent associations between childhood survival and the number of older siblings (see §2f), as found in other studies [19]. Accounting for the total number of older siblings also automatically controls for potential effects of being firstborn on survival (as they have zero older siblings), and we therefore did not separately incorporate a firstborn variable [69]. We excluded twins from the analyses as focal individuals (*n* = 257) owing to the differences in survival and other factors associated with twins [70,71].

### (e) Parental variables

Following Evans *et al*. [72], we used the father’s occupation as a proxy for a family’s socioeconomic status. Occupations were standardized following the Historical International Standard Classification of Occupations (HISCO) [73] and assigned a numeric value for socioeconomic status using the historical social stratification scale (HISCAM) [74]. HISCAM uses records of intergenerational interactions and marriages between different occupations from 1800 to 1938 across northern Europe and Canada to assign different occupations a socioeconomic status ranging from 1 to 100 [74]. In cases where multiple occupations were present (*n* = 472, with up to eight occupations), we used the occupation with the highest HISCAM. A HISCAM score was assigned to 2333 fathers with recorded occupations, providing measures of socioeconomic statuses that ranged from 39.9 to 99 on an interval scale (servant to lieutenant, respectively), resulting in 7438 focal offspring (58%) with a known family socioeconomic status that could be used to control for potential positive effects of wealth and social status on childhood survival [75].

We determined whether the mother and/or father died during the focal individual’s first 5 years (918 and 524 cases, respectively) to control for the negative impact this may have on offspring survival [76,77]. Additionally, both the mother’s and father’s ages at birth were included to account for potential parental age effects on early-life survival [58,78]. Finally, we included the number of grandparents alive in the first 5 years of the child’s life to control for the positive effect they may have on their grandchildren’s survival [56].

### (f) Statistical analyses

We modelled the association between the number of older siblings and childhood survival using generalized linear mixed models (GLMMs) with the *glmer* function from *lme4 1.1.31* [79] in R 4.2.2 [80], with a binomial error and logit-link. This approach suited our aim of estimating net childhood survival across a structured set of models, which we describe below. Given the binary outcome variable and the need for the inclusion of random intercepts and slopes, we adopted a GLMM approach, which can readily accommodate these (also see [19]).

We first fitted a baseline model (*m1*) estimating the association between childhood survival and the total number of older siblings. We then ran separate models increasing in complexity, decomposing the number of older siblings into further categories. First, we split the number of older siblings into those that were alive or deceased at the focal individual’s time of birth (*m2*). We then decomposed these categories further into the number of living or deceased older siblings born close (< 5 years) and far in age (  years) from the focal individual (*m3*). Finally, we further decomposed these categories into the number of older living and deceased brothers and sisters born close (< 5) and far (≥ 5) in age (*m4*).

To control for biologically meaningful and potentially confounding variables affecting childhood survival, all models included the following categorical fixed effects: the sex of the focal individual, the birth parish (either Linthal or Elm) and whether the focal individual experienced the death of their mother or father during childhood. As linear covariates, we included socioeconomic status, mother and father age, and the number of grandparents alive at the date of birth of the focal individual. Squared terms were also added for parental age effects but removed, least significant first, if non-significant to aid interpretation of the first-order effects. We modelled variation in childhood survival among families and across 5 year parish-specific birth cohorts by including both as random effects. Additionally, we fitted random slopes for each sibling variable to quantify their effect within families [81]. We also tested for interactions between the sex of the focal individual and all variables relating to the numbers of siblings of different categories in all models to test if any effects of sibling presence were sex-dependent. Models were fitted using only individuals informative for all predictors, leaving 2941 focal individuals, including 1454 females and 1487 males.

Significance of all fixed effects was determined using likelihood ratio tests (LRTs), using the *drop1* function (*stats 4.2.2* [80]). Interactions were removed if non-significant (stepwise, highest *p*-values first) to improve interpretability of the results, but otherwise all predictors were retained in the model irrespective of their statistical significance. Results including non-significant interactions for each model are shown in electronic supplementary material, tables S2, S5 and S6. If an interaction with the focal individual’s sex was significant, a post hoc test was conducted using *emmeans 1.8.4-1* [82] to determine whether the association between childhood mortality and the variable was statistically significant within each sex. DHARMa *0.4.6* was used for model diagnostics [83]. Specifically, the KS test and QQ plots were used to examine whether residuals followed a normal distribution, and we tested for overdispersion, heteroscedasticity and outliers. None of these tests revealed violations of model assumptions. Collinearity between variables was low for most variables across all models (variance inflation factor < 5) and only surpassed 4 for the maternal age variable (assessed using *vif* from *car 3.1.1* [84]). We used *ggeffects 1.1.5* to predict the differences in survival between children with different numbers of siblings (comparing 0 versus 2), with all other predictors held at their reference for categorical predictors and at the mean for numeric predictors [85]. For data visualization, we used *ggplot2 3.4.1* and *ggpubr 0.5.0* [86,87]. To aid model convergence, the ‘bobyqa’ optimizer was used, and all non-categorical predictor variables were mean-centred and scaled to a standard deviation of 1.

## 3. Results

**Figure 1.**
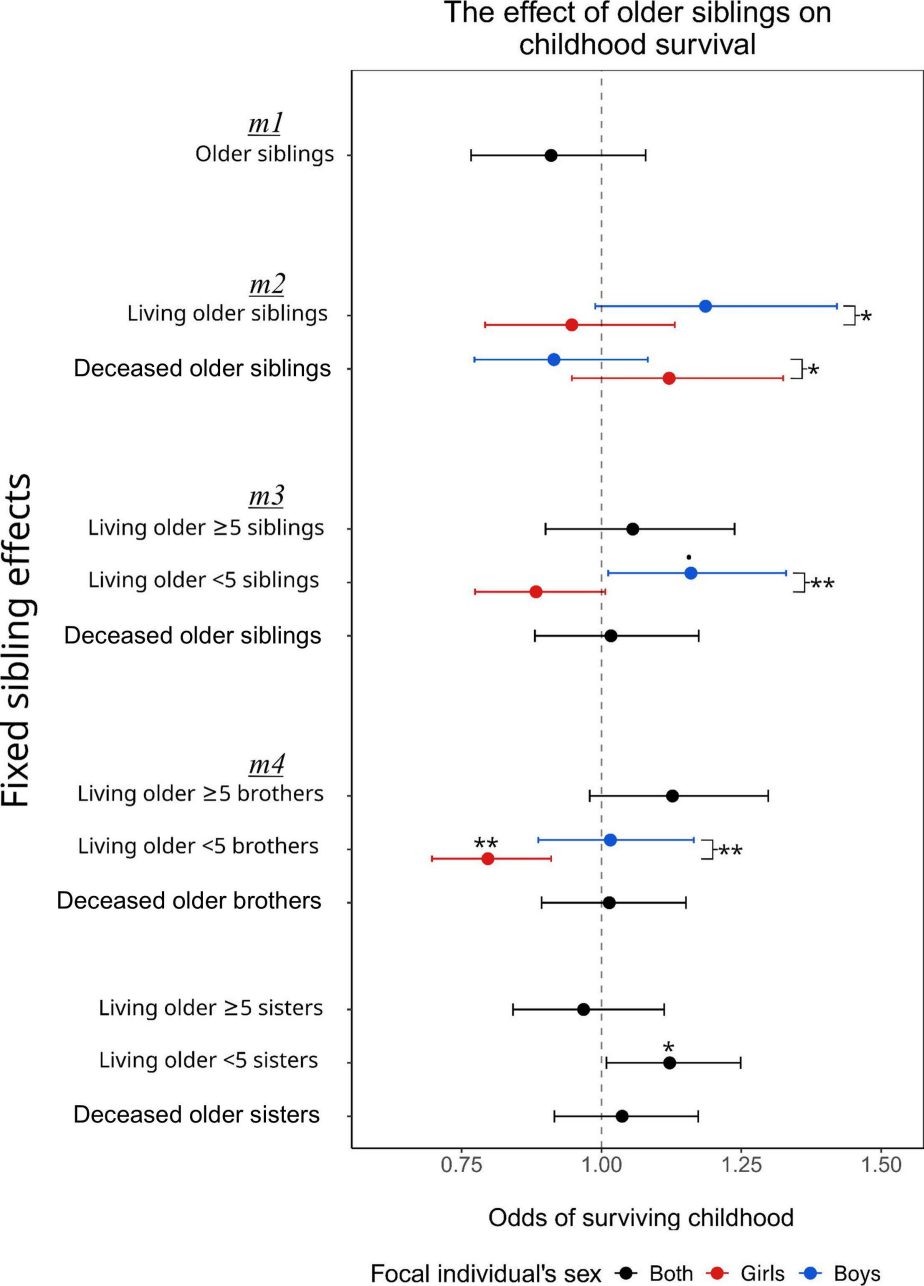
Associations between the total number of older siblings and childhood survival (*m1*), and then when decomposed by their survival status (*m2*), age (*m3*) and sex (*m4*). On the *x*-axis are the respective odds ratios of surviving, and error bars show the 95% confidence intervals. *p*-values are shown as: •*p* < 0.1, *p < 0.05 and **p < 0.01. We present sex-specific odds ratios and confidence intervals (females = red; males = blue) when associations were sex-dependent. The significance of sex-specific effects was taken from post hoc tests.

Overall, 73% of the focal individuals in our study survived childhood (*n* = 2141/2941). In our baseline model (*m1*), childhood survival was not associated with the number of older siblings (odds ratio = 0.91, 95% CI = [0.77–1.08], *p* = 0.282; figure 1; electronic supplementary material, table S1). This association did not vary across families (*p* = 0.157, electronic supplementary material, table S1) and was not dependent on the sex of the focal individual (*p* = 0.551; electronic supplementary material, table S2). However, childhood survival was higher for individuals born in Elm than in Linthal (0.674 [0.527 - 0.861], *p* = 0.003; electronic supplementary material, table S1); for individuals with mothers of intermediate age who survived the first 5 years of the focal individual’s life (0.891 [0.823–0.965], *p* = 0.005, and 1.923 [1.068–3.461], *p* = 0.032, respectively, electronic supplementary material, table S1); and for individuals with older fathers (1.206 [1.022–1.422], *p* = 0.027; electronic supplementary material, table S1). Childhood survival was not associated with paternal survival across the first 5 years of the focal individual’s life, their fathers’ socioeconomic status, their sex or the number of grandparents alive at the time of their birth (*p* > 0.05; electronic supplementary material, table S1). Finally, childhood survival varied significantly among families but not among birth cohorts (*p* < 0.001 and *p* = 0.120; respectively, electronic supplementary material, table S1).

However, dividing focal individuals by their sex and dividing their older siblings by whether they were alive when the focal individual was born (*m2*) revealed associations between childhood survival and the total number of both living and deceased older siblings that were dependent upon the sex of the focal individual (*p* = 0.018 and *p* = 0.026, respectively; electronic supplementary material, table S3; figure 1). For example, having two living older siblings versus zero increased survival from 73% [51–88%] to 76% [56–89%] for males but decreased the survival probability of females from 79% [60–90%] to 78% [0.58–0.90%]. Conversely, having two deceased older siblings versus none was associated with a decrease in survival in boys, from 78% [57–90%] to 75% [53–88%], but an increase in girls from 77% [56–90%] to 80% [61–91%]. However, within boys and girls, the effect of the number of (living or deceased) older siblings was not statistically significant (the number of living older siblings on boys: *p* = 0.127 and on girls: *p* = 0.794; the number of deceased older siblings on boys: *p* = 0.515 and on girls: *p* = 0.334; figure 1).

We then further divided the number of living older siblings into those close in age (< 5 years old when the focal individual was born) or far in age (≥ 5 years of age) (*m3*, electronic supplementary material, table S4). In this model, neither the number of deceased nor living older (≥ 5) siblings affected childhood survival (1.017 [0.881–1.174], *p* = 0.817, and 1.056 [0.900–1.238], *p* = 0.516, respectively, electronic supplementary material, table S4; figure 1) and these associations were not dependent upon the sex of the focal individual (*p* = 0.080 and *p* = 0.386, respectively; electronic supplementary material, table S5). The association between childhood survival and the number of living older (< 5) siblings was however dependent on the sex of the focal individual (*p* = 0.003; electronic supplementary material, table S4): having two living older (< 5) siblings versus none increased the survival of boys from 73% [51–88%] to 80% [60–91%] and decreased the survival of girls from 80% [61–91%] to 75% [53–89%]. However, although significantly different from each other, neither of these odds ratios was significantly different from one (boys: *p* = 0.065; girls: *p* = 0.124; figure 1).

Finally, we split the number of living older siblings close and far in age into brothers and sisters (*m4*). This revealed a sex-dependent association between the childhood survival and the number of older (< 5) brothers (*p* = 0.007; table 1): girls with two rather than no older (< 5) brothers had a survival probability of 69% [44–86%] versus 82% [63–0.92%] (*p* = 0.002; figure 1), but boys’ survival was not associated with the number of older (< 5) brothers (77% [55–90%] versus 77% [55–91%], *p* = 0.966; figure 1). Individuals with more living older (≥ 5) brothers had marginally higher survival (*p* = 0.105; table 1; figure 1), such that the survival probability of individuals with two living older (≥ 5) brothers versus none increased from 78% [58–91%] to 82% [63–93%], regardless of their sex (*p* = 0.140, electronic supplementary material, table S6). Having more living older (< 5) sisters was also positively associated with an individual’s childhood survival (*p* = 0.029; table 1; figure 1), with the survival probability of individuals with two older (< 5) sisters versus none increasing from 78% [58–91%] to 84% [66–94%], regardless of the focal individual’s sex (*p* = 0.124, electronic supplementary material, table S6). However, childhood survival was not associated with the number of living older (≥ 5) sisters or the number of deceased older brothers or sisters (*p* = 0.656, *p* = 0.831 and *p* = 0.562, respectively; table 1; figure 1). The associations of the sex- and age-specific sibling variables with childhood survival showed no variation across cohorts (*p* > 0.05; table 1), but there remained unexplained variation in childhood survival between families (*p* < 0.001; table 1).

**Table 1.** Logistic GLMM showing the association between the childhood survival and the number of older siblings from *m4* (*n* = 2941), in which we decomposed the number of older siblings into deceased and living older sisters and brothers born close and far in age (≥ 5) to the focal individual and present their associations with childhood survival, together with the other predictors of survival included in the model. Odds ratios, 95% confidence intervals, variation explained by random effects and *p*-values from LRT tests are presented. Interactions are shown with a multiplication symbol (×). Odds ratios significantly different from 1 are in bold. *p < 0.05; **p < 0.01; ***p < 0.001.

	childhood survival (<5)		
fixed effects	odds ratio	95% CI	*p*‐value
*intercept*	3.879	1.441−10.442	—
sex (male)	0.850	0.712−1.016	—
the number of living older (≥ 5) brothers	1.127	0.979−1.298	0.105
the number of living older (< 5) brothers	0.797	0.697−0.910	—
the number of deceased older brothers	1.014	0.893−1.151	0.831
the number of living older (≥ 5) sisters	0.968	0.842−1.112	0.656
the number of living older (< 5) sisters	1.122*	**1.009−1.249**	**0.029**
the number of deceased older sisters	1.037	0.916−1.173	0.562
mother age	0.826*	**0.687−0.993**	**0.042**
mother age²	0.868**	**0.797−0.946**	**0.001**
father age	1.146	0.969−1.356	0.112
maternal survival	2.016*	**1.107−3.672**	**0.023**
paternal survival	0.634	0.288−1.395	0.249
socioeconomic status	0.927	0.831−1.034	0.175
parish (linthal)	0.652**	**0.505−0.842**	**0.002**
the number of grandparents living at birth	0.985	0.875−1.109	0.805
sex (male) × the number of living older (< 5) brothers	1.276**	**1.070−1.521**	**0.007**

random effects	variance	*p*‐value
cohort (intercept)	0.204	0.098
family (intercept)	0.589***	**<0.001**
the number of living older (≥ 5) brothers | family (random slope)	0	1
the number of living older (< 5) brothers | family (random slope)	0	1
the number of deceased older brothers | family (random slope)	0.015	0.993
the number of living older (≥ 5) sisters | family (random slope)	0.261	0.174
the number of living older (< 5) sisters | family (random slope)	0.103	0.805
the number of deceased older sisters | family (random slope)	0	1

The associations between childhood survival and all other variables were consistent across models (*m1–m4*; table 1; electronic supplementary material, tables S1–S6), except for the association between father age and childhood survival being non-significant in *m2* (*p* = 0.088; electronic supplementary material, table S3), *m3* (*p* = 0.098; electronic supplementary material, table S4) and *m4* (*p* = 0.112; table 1).

## 4. Discussion

Although at first sight childhood survival did not appear to be associated with the total number of older siblings (figure 1), distinguishing between siblings on the basis of their survival status, sex, and age difference with the focal individual revealed both positive and negative associations. Thus, these results argue against exclusively positive associations between the presence of older siblings and survival [25]. Instead, they suggest that some siblings provide benefits and others are detrimental, and that these also depend on the sex of the focal individual. Thereby, we show that siblings are an important—but complex and context-dependent—component of the early-life environment.

Several of our results are consistent with a parental preference for sons. First, we found that girls—but not boys—with more living older brothers close in age had reduced childhood survival. Having older brothers who are close in age could be detrimental to younger siblings of either sex due to their larger size and greater energetic demands [40–42], but the fact that this cost was limited to females suggests that parents might have tried to shield their younger sons from these costs. This is consistent with the male-favoured or gendered resource dilution model [33,49]. We also found that having more living older siblings increased childhood survival of boys more than of girls, whereas having more dead older siblings benefitted females more. Although these sex differences became weaker with further decomposition (electronic supplementary material, tables S4 and S6; table 1), this suggests that help provided by siblings benefitted boys more than girls and that deaths increased the chances of survival for girls. Evidence of male preference is in line with the patrilineal inheritance in our study system, which could have motivated differential parental investment, prioritizing boys [88]. However, unlike other studies [16,19,33], we did not find lower childhood survival of those born with many same-sex older siblings, and hence no evidence for same-sex sibling competition (electronic supplementary material, table S4), illustrating how siblings’ effects on survival can vary across cultural and temporal contexts (also see below).

In contrast to the negative association with the number of older brothers close in age, the number of older sisters close in age was positively associated with the survival of their younger brothers and sisters. This differs from findings by [33], who found the association between the total number of older sisters to be positive for males but negative for females—perhaps due to same-sex competition. Similarly, Nitsch *et al*. [19] found a positive association of the total number of older sisters with male survival but none with female survival. While these studies have suggested the positive association may be due to older sisters helping younger siblings, this cannot explain why we did not find similar associations for older sisters more distant in age. Alternatively, positive associations between the number of older sisters close in age and survival may be due to individuals with more older sisters (< 5) being less likely to have older brothers (< 5), or to suffer the negative consequences of being a firstborn [89]. However, firstborn effects on survival are nuanced [69] and, overall, these associations illustrate that while siblings may have detectable effects on early-life survival, these are difficult to interpret and likely multifaceted, and at the moment should be interpreted with care.

Albeit weak, we found some evidence that older brothers further away in age are positively associated with childhood survival, suggesting they helped younger siblings survive (figure 1). No such associations were found for older sisters. This is similar to associations found by Nitsch *et al*. [19], who hypothesized that older brothers might have helped the productivity of family farms through providing labour. Future studies could test this by comparing farm-owning versus non-farm-owning families. In our population, there was also a strong wage gap between males and females, meaning older brothers may have benefitted their families’ resources through income, while older sisters had a more limited ability to do so [90]. Further, as potential inheritors, older brothers may have stayed with their families for longer than sisters, giving them greater opportunity to influence the development of their younger siblings than older sisters. The lack of an upper age limit for siblings further away in age (≥ 5) may partly explain the absence of a detectable effect of older brothers and sisters in these categories, as it is possible that some siblings in these categories had reached an age at which they no longer influenced their younger siblings, for instance, by having left the household. However, 99.6% of individuals in the category of siblings further away in age (≥ 5) had an age difference of less than 25 years at the birth of their younger sibling, which is also the median age of first reproduction. Since the first reproduction most likely coincided with marriage and household departure, it is likely that the majority of these siblings were still residing in the household and thus capable of influencing their younger siblings’ survival. Finally, the absence of an association with the number of dead brothers does not contradict the observed positive effect of living brothers, which may further reflect a beneficial influence, although the precise mechanism remains unknown.

Albeit valuable, comparisons to other studies are hampered by differences in the geographical location, time period and approaches used to analyse survival. With the exception of Fox *et al*. [16], who report sibling associations in Krummhörn in Germany, most other studies were conducted in areas considerably further away from Switzerland, where differences in cultural norms, household structures, occupational patterns and living conditions may all have contributed to variation in associations of older siblings with survival (Finland [19]; Quebec [16]; Taiwan [33]; Malawi [45]; Gambia [46]; Morocco [36]; Bolivia [37]). Similarly, the historical time period under study varies substantially among studies (1906‒1945 [33]; 1997 [45]; 1950−1974 [46]; 1984 [36]; 1998–1999 [37]). Finally, our focus on older sibling age and sex, in combination with the focal individual’s sex, makes direct comparison with other studies challenging, with only Riswick & Hsieh [33] adopting a similar approach. Overall, our study fills a gap in the literature by offering new insights into sibling associations with childhood survival across a previously unexamined combination of cohort and historical time period, while highlighting the role of age and sex in shaping these associations.

Our study has some limitations. First, its observational nature makes causal inference challenging, especially when the underlying mechanisms mediating the survival effects of siblings are not well understood. Isolating associations with sibling number in GLMMs (or comparable approaches such as cox-proportional hazard models) in human populations is often challenging, and other variables (e.g. parental age) may be confounded with birth order effects. To delve further into these complexities and build on our findings, future studies could explore the ability of structural equation models or event history analysis to provide a higher-resolution insight into some of the described associations [91]. Second, although we found evidence for older brothers and sisters influencing the early-life survival of their younger siblings, these associations were close to the threshold of statistical significance. This illustrates the difficulties of disentangling these effects in historical human populations, even with very large sample sizes. Related to this, we did not explicitly control for the effects of younger siblings, which could also affect childhood survival. However, as opposed to younger siblings, older siblings can affect early-life survival from the moment of birth and are therefore probably more important. Furthermore, simultaneously including the number of younger siblings introduces additional complexity to what are already complex and data-hungry models. Finally, the number of younger siblings is likely to correlate with the number of older siblings, making it difficult to reliably separate their effects. Nevertheless, exploring the effects of younger and older siblings in tandem would be an avenue for future studies to explore.

In this study, we provided a comprehensive decomposition of how older siblings shape survival in early life. Thereby, our study fills a gap in the literature by offering new insights into sibling associations with childhood survival across a previously unexamined community and historical time period. We emphasize the need to consider interactions with the sex, survival and age of siblings, which are mediators of both the strength and the direction of sibling-related associations. Overall, we provide a rare insight into how siblings can shape survival in a long-lived species, suggesting signals consistent with both cooperative and competitive interactions mediated by biological and cultural factors that align with the gendered resource dilution model. Thereby these results show that siblings are an important component of the early-life environment.

## Data Availability

Code and data necessary for reproducing results are available at [92]. Supplementary material is available online [93].
